# Pre-Analytical Stability of Mycophenolic Acid and Its Glucuronide in Saliva Samples Stored in Cotton Salivette^®^ Devices Before Centrifugation

**DOI:** 10.3390/pharmaceutics18091106

**Published:** 2026-09-02

**Authors:** Grzegorz Szynkaruk, Julia Kerner, Ahata Nelipovich, Kacper Osuch, Maria Miotk, Agata Bartkowiak, Joanna Sobiak

**Affiliations:** 1 The Student Scientific Society of Poznan, University of Medical Sciences, 5 Rokietnicka Street, 60-806 Poznan, Poland; 2 Department of Physical Pharmacy and Pharmacokinetics, Poznan University of Medical Sciences, 3 Rokietnicka Street, 60-806 Poznan, Poland

**Keywords:** mycophenolic acid, saliva, saliva collection, stability, therapeutic drug monitoring, mycophenolate mofetil, pediatrics, mycophenolic acid glucuronide

## Abstract

**Background/Objectives**: Saliva may provide a non-invasive alternative to blood sampling for therapeutic drug monitoring (TDM) of mycophenolic acid (MPA) in children. This study assessed MPA and, secondarily, mycophenolic acid glucuronide (MPAG) in non-centrifuged Salivette^®^ devices to determine an acceptable interval between saliva collection and laboratory processing. **Methods**: Phosphate-buffered saline (PBS), artificial saliva, and saliva from five healthy adult volunteers were externally spiked with MPA and MPAG at 5 and 500 ng/mL and applied to Salivette^®^ cotton swabs. Samples were stored for 12, 24, and 48 h at 22 °C and 6 °C and analyzed by liquid chromatography-tandem mass spectrometry. Percentage deviations from nominal concentration within ±15% were considered acceptable. **Results**: In PBS, both analytes met the acceptance criterion for up to 24 h under both conditions. In artificial saliva, both met the criterion for at least 24 h and, under some conditions, for 48 h. In human saliva, all volunteer-level MPA results met the criterion after 12 h at both temperatures, whereas MPAG did not consistently meet it. Mean MPA deviations across volunteers ranged from −10.8 to 1.9% at 22 °C and from −12.3 to 2.4% at 6 °C. **Conclusions**: MPA met the acceptance criterion after 12-h in non-centrifuged Salivette^®^ devices, supporting the feasibility of a 12-h pre-centrifugation interval under comparable conditions. Prompt centrifugation and processing are advisable when MPAG determination is required. Confirmation using incurred post-dose saliva from pediatric patients receiving mycophenolate mofetil is required.

## 1. Introduction

Mycophenolic acid (MPA), the active moiety of mycophenolate mofetil (MMF), is frequently used in the treatment of nephrotic syndrome. Therapeutic drug monitoring (TDM) of MPA has been recommended in this indication, although it is not routinely implemented. MPA exhibits complex pharmacokinetics, including (1) a poor relationship between the pre-dose trough concentrations and the area under the concentration-time curve from 0 to 12 h (AUC_0–12_), (2) secondary concentration peaks resulting from enterohepatic recirculation, and (3) extensive plasma protein binding (97–99%) [1].

Saliva has emerged as a promising noninvasive biological matrix for TDM. It contains compounds derived from the systemic circulation, primarily through mucosal transudation and gingival crevicular fluid. Saliva is easily accessible and inexpensive and can be collected without causing substantial stress or discomfort to the patient. Moreover, saliva collection does not require trained healthcare personnel and may be performed at home by the patient or caregiver [2]. For some drugs, salivary concentrations may reflect the pharmacologically active unbound fraction in plasma [3]. Nevertheless, published findings regarding the relationship between salivary MPA concentrations and total or unbound plasma concentrations remain inconsistent. Although several studies have reported significant correlations [4,5,6,7], others have failed to confirm these findings [8,9]. Salivary TDM of MPA has therefore not yet been introduced into routine clinical practice, and further studies are required to standardize sampling, handling, and sample-processing procedures [1].

This approach may be particularly valuable in pediatric MPA monitoring because reliable pharmacokinetic assessment is based primarily on exposure over the dosing interval rather than on a single trough concentration and may therefore require repeated sampling [1,10,11,12]. Home-based or remote saliva collection could facilitate concentration–time profiling and the future development of limited-sampling strategies [13,14]. However, these applications require adequate control of the pre-analytical phase. Delayed centrifugation, prolonged contact with the absorbent material, and inappropriate storage conditions may alter measured concentrations, and consequently affect pharmacokinetic profiles and AUC estimates [15,16]. Such pre-analytical variability may also weaken apparent exposure–response relationships and compromise future dose-individualization strategies. Establishing acceptable handling conditions for non-centrifuged Salivette^®^ devices is therefore an essential prerequisite for the future implementation of saliva-based MPA TDM in pediatric nephrology.

Our previous studies addressed complementary but distinct analytical and clinical questions. First, we validated a high-performance liquid chromatography method with fluorescence detection (HPLC–FLD) for salivary MPA determination and evaluated its stability under several post-collection storage and processing conditions [17]. Subsequently, we developed and validated a liquid chromatography–tandem mass spectrometry (LC–MS/MS) method for the simultaneous determination of MPA and mycophenolic acid glucuronide (MPAG) in saliva [18]. That study assessed the stability of both analytes in recovered saliva, processed samples, and analytical samples and included a short-term recovery test following a 5-min incubation on Salivette^®^ cotton swabs to simulate the collection procedure. Under those conditions, the results did not indicate substantial short-term analyte loss associated with the cotton swabs. In both validation studies, however, the Salivette^®^ devices were centrifuged immediately after saliva collection, and stability was therefore evaluated after recovery of saliva from the device rather than during prolonged contact with the cotton swab. In our subsequent clinical study of plasma–saliva relationships in children with nephrotic syndrome [12], the Salivette^®^ devices were also centrifuged immediately after collection.

Consequently, prolonged storage of saliva-loaded cotton Salivette^®^ devices before centrifugation remained uninvestigated. This pre-centrifugation interval is particularly relevant to outpatient, decentralized, or home-based sampling, in which samples may be collected at predefined time points after drug administration and transported to the laboratory before further processing. Establishing whether MPA and MPAG concentrations remain within a predefined acceptance limit during this period is, therefore, an important prerequisite for the future implementation of saliva-based TDM.

The primary aim of this study was to evaluate MPA stability during predefined intervals between saliva collection using cotton Salivette^®^ devices and laboratory processing and to identify the longest tested pre-centrifugation storage interval at which MPA met the predefined acceptance criterion. As a secondary objective, the stability of MPAG was evaluated under the same conditions. Stability was first assessed in phosphate-buffered saline (PBS) and artificial saliva and subsequently evaluated in spiked saliva collected from healthy adult volunteers. This study addressed a clinically relevant pre-analytical step that has not been investigated previously. The findings may help inform sample-handling procedures for future studies of saliva-based MPA TDM in children. However, the present study represents an enabling pre-analytical assessment rather than a clinical validation of salivary MPA monitoring or individualized MMF dosing.

## 2. Materials and Methods

### 2.1. Chemicals and Reagents

All solvents and reagents were of liquid chromatography–mass spectrometry (LC–MS) grade. Artificial saliva, MPA, and deuterated MPA were purchased from Sigma-Aldrich (Steinheim, Germany). MPAG was obtained from LGC Standards (Toronto Research Chemicals, Toronto, ON, Canada). Methanol, acetonitrile, and water (hypergrade for LC–MS) were purchased from Merck (Darmstadt, Germany). PBS (pH 7.4) was purchased from Chempur^®^ (Piekary Śląskie, Poland). Salivette^®^ devices with cotton swabs were obtained from Sarstedt (Nümbrecht, Germany).

### 2.2. LC-MS/MS Analysis

The concentrations of MPA and MPAG were determined using a previously validated LC–MS/MS method [18]. Matrix-matched calibration curves were prepared separately in PBS, artificial saliva, and blank human saliva obtained from healthy volunteers. For each calibration standard, 100 µL of the corresponding blank matrix was mixed with 50 μL of the appropriate MPA and MPAG working solution and 50 μL of the MPA-d3 internal standard solution. For each study sample, 100 µL of PBS, artificial saliva, or human saliva previously spiked with MPA and MPAG and stored under the investigated conditions was mixed with 50 µL of methanol and 50 µL of the MPA-d3 internal standard solution. After mixing, the samples were evaporated to dryness at 45 °C for 2 h in a centrifugal vacuum concentrator (Eppendorf, Germany). The dry extract was reconstituted in 100 μL of methanol-water (1:4, *v*/*v*), with each solvent containing 0.1% formic acid before mixing. The samples were transferred to Eppendorf tubes, centrifuged twice for 10 min at 16,000× *g*, and 10 μL was injected into the LC–MS/MS system. This part of the study was conducted with the support of the Center of Innovative Pharmaceutical Technology, Poznan University of Medical Sciences. The previously validated method was linear over the concentration range of 2–500 ng/mL for both analytes, with the lower limit of quantification (LLOQ) of 2 ng/mL for both salivary MPA and MPAG. Across the four validated QC levels, within-run and between-run accuracy ranged from 92.5% to 106.2% for MPA and from 95.1% to 114.7% for MPAG. Precision did not exceed 14.6% for MPA or 12.0% for MPAG. In the matrix-effect assessment performed using saliva from seven healthy volunteers at 5 and 500 ng/mL, accuracy ranged from 90.6% to 112.1% for MPA and from 95.6% to 113.7% for MPAG, while precision did not exceed 9.0% and 10.1%, respectively. Short-term apparent recovery following a 5-min incubation on Salivette^®^ cotton swabs at 37 °C ranged from 90.9% to 101.6% for MPA and from 100.1% to 100.7% for MPAG, with coefficients of variation not exceeding 9.5% [18]. No formal revalidation or matrix-effect assessment was performed in PBS or artificial saliva for the present study. Each study sample was therefore quantified using a matrix-matched calibration curve prepared in the corresponding matrix.

### 2.3. Saliva Collection from Healthy Volunteers

Saliva was collected from five healthy volunteers with a mean age of 42 ± 5 years (one man and four women) using the passive drool method. Before collection, the volunteers rinsed their mouths with water and refrained from eating for at least 1 h. They subsequently expectorated saliva into a sterile container for approximately 10 min. The mean ± standard deviation (SD) values for the pH and volume of saliva collected for the 12-h storage experiment were 7.32 ± 0.26 and 8.93 ± 2.81 mL, respectively; the corresponding values for the 24-h storage experiment were 7.20 ± 0.19 and 7.20 ± 1.89 mL, respectively. The saliva samples were processed immediately after collection. Saliva from each volunteer was processed separately and spiked independently. For each combination of volunteer, quality control (QC) level, and storage condition that was evaluated, three separately prepared aliquots from the same saliva sample were used as technical replicates. The number of volunteers was selected pragmatically for this exploratory laboratory study to provide an initial assessment across several independent human saliva matrices. No formal sample-size calculation was performed because the study was not designed to characterize population-level variability. All procedures involving human participants were conducted in accordance with the ethical standards of the institutional and national research committees, as well as the 1964 Helsinki Declaration and its later amendments or comparable ethical standards. The study was approved by the Bioethical Committee at Poznan University of Medical Sciences (Decision 737/25). Written informed consent was obtained from volunteers prior to enrollment.

### 2.4. Sample Preparation and Stability Assessment

The pre-centrifugation stability of MPA and MPAG was evaluated in three matrices: PBS, artificial saliva, and human saliva obtained from five healthy volunteers. Samples were prepared at nominal MPA and MPAG concentrations of 5 and 500 ng/mL, corresponding to the low- and high-QC levels of the LC-MS/MS method, respectively. The high-QC concentration corresponded to the upper limit of quantification of the validated method. These two QC levels were selected to assess pre-analytical stability at low and high concentrations within the validated analytical range and to explore whether pre-analytical performance differed according to analyte concentration. For each matrix and concentration level, the prepared samples were divided into three aliquots, each of which was applied to a separate Salivette^®^ cotton swab. A total volume of 580 µL was applied to each low-QC sample and 600 µL to each high-QC sample. After application of the spiked matrix, the cotton swabs were incubated for 5 min at 37 °C before storage. The 5-min interval was selected for consistency with the short-term swab-recovery experiment performed in our previous method-validation study [18]. This interval was longer than the 2-min oral collection time recommended by the manufacturer and was used as a standardized, slightly prolonged contact period at approximately oral temperature rather than as an exact simulation of routine saliva collection. The devices were subsequently stored either at room temperature (approximately 22 °C) or under refrigeration (approximately 6 °C). Samples were protected from light during storage. The Salivette^®^ tubes were closed with their original caps but were not additionally sealed, for example, with parafilm, to reflect the intended home-storage conditions. The refrigerator and laboratory temperatures were checked using a thermometer. These conditions were intended to represent refrigerated storage and moderate indoor room-temperature storage.

PBS and artificial saliva samples were evaluated after 12, 24 and 48 h of storage. Human saliva samples were evaluated after 12 and 24 h; the 48-h interval was not evaluated in human saliva. At each applicable time point, the Salivette^®^ devices were centrifuged at 1000× *g* for 2 min according to the manufacturer’s instructions. The exact total volume recovered from each device was not recorded. However, at least 100 µL was recovered from every evaluated device, and 100 µL was used for LC–MS/MS analysis. Samples were analyzed against calibration curves prepared using freshly spiked standards, as described in Section 2.2. Not all volunteers contributed evaluable samples at the 24-h time point. After the initially evaluated 24-h samples produced results outside the predefined acceptance range, the remaining 24-h experiments were not performed. The subsequent 12-h assessment was conducted using newly collected saliva samples. Therefore, the missing 24-h observations represent experiments that were not performed rather than analytical failures. Evaporative loss was not directly assessed. Although short-term apparent recovery had previously been evaluated in human saliva [18], recovery was not compared among PBS, artificial saliva, and human saliva or evaluated after prolonged storage of non-centrifuged devices.

An additional exploratory assessment of frozen storage was performed using artificial-saliva filtrates obtained after centrifugation following 12 or 24 h of pre-centrifugation storage in Salivette^®^ devices. The filtrates were transferred to separate tubes and stored at −80 °C for four months. After thawing, 100 µL aliquots were analyzed using the LC–MS/MS procedure described above. This experiment was limited to artificial saliva, which served as a standardized model matrix, because the volume of human saliva obtained by passive drool was insufficient to perform the additional long-term assessment.

The use of low and high QC levels and the operational acceptance criterion was informed by the International Council for Harmonisation guideline M10 on bioanalytical method validation and study sample analysis [19]. For each analyte and QC level, the mean back-calculated concentration obtained from three technical replicates was required to be within 85–115% of the nominal concentration. Results were expressed as percentage deviation from the nominal concentration; therefore, the predefined acceptance range corresponded to ±15%. A contemporaneous time-zero sample consisting of freshly spiked matrix applied to the Salivette^®^ cotton swab and centrifuged immediately was not included. Consequently, the study did not constitute a formal stability validation fully consistent with the ICH M10 design. The results represent deviations from the nominal QC concentrations rather than changes relative to a measured baseline. The observed deviations therefore cannot be attributed exclusively to storage-related changes and may also reflect immediate analyte loss, incomplete recovery, matrix-dependent analytical effects, variability in sample preparation, or other pre-analytical factors.

The analysis of the human-saliva experiments was descriptive. Each of the five healthy adult volunteers was treated as one independent biological observation. For each combination of volunteer, storage condition, analyte, and QC level, the mean ± SD was calculated from three technical replicates prepared from the same saliva sample. The technical replicates were averaged to obtain a single volunteer-level result and were not treated as independent biological observations. Because the replicates included separate preparation, storage, recovery, and analysis, they reflected within-condition technical variability in the overall procedure rather than exclusively instrumental analytical variability. A volunteer-level result was considered to meet the predefined acceptance criterion when its mean percentage deviation from the nominal concentration was within ±15%. Missing observations were not imputed. For each condition, the number of evaluable volunteers meeting the criterion was reported as n/N. Volunteer-level percentage deviations were additionally summarized for each combination of analyte, storage temperature, storage time, and QC level using the median and range (Supplementary Table S1). No inferential statistical tests or confidence intervals, or formal comparisons between storage temperatures, storage times, or QC levels were performed because of the exploratory study design, the small and partly unbalanced number of independent biological observations, and the absence of a prespecified hypothesis-testing framework.

## 3. Results

First, stability was assessed in PBS. For both MPA and MPAG, the predefined acceptance criteria were met for up to 24 h at both room temperature and under refrigerated conditions. After 24 h of storage at room temperature, the mean percentage deviations ± SD for MPA at the low and high QC levels were −4.7 ± 7.0% and −6.4 ± 0.8%, respectively. For MPAG, the corresponding values were 2.0 ± 4.4% at the low QC level and −5.2 ± 0.5% at the high QC level. After 24 h of refrigerated storage, the mean percentage deviations for MPA were −8.0 ± 4.4% at the low QC level and −5.2 ± 5.2% at the high QC level. The corresponding values for MPAG were −5.1 ± 6.0% and −0.9 ± 0.4%, respectively. After 48 h of storage, the predefined acceptance criterion was not met for either analyte under either storage condition (Table 1).

Next, stability was assessed in artificial saliva. At room temperature, MPA met the predefined acceptance criterion for up to 24 h, whereas MPAG met the criterion for up to 48 h. After 48 h at room temperature, the mean percentage deviation for MPA at the low-QC level was −15.5 ± 6.8%, corresponding to a mean concentration below 85% of the nominal concentration and therefore outside the predefined acceptance range (Table 2). Under refrigerated conditions (6 °C), both MPA and MPAG met the predefined acceptance criterion for up to 48 h (Table 2).

After four months of storage at −80 °C, MPA showed marked negative deviations from the nominal concentration, particularly at the low-QC level and in filtrates obtained after 24 h of pre-centrifugation storage. The predefined acceptance criterion was met only for the 500 ng/mL MPA samples obtained after 12 h of pre-centrifugation storage at both initial storage temperatures. MPAG generally showed smaller mean deviations than MPA; however, the predefined acceptable criterion was not met under all conditions, and substantial variability was observed at the low-QC level. Thus, acceptable results were not consistently demonstrated for the combined procedure involving delayed centrifugation followed by four months of storage at −80 °C. Detailed results are presented in Supplementary Table S2.

Finally, the stability assessment was performed in saliva obtained from five healthy adult volunteers (Table 3). Each volunteer-level result represented the mean ± SD of three technical replicates, whereas the denominators reported below refer to independent evaluable volunteers. After 12 h of storage, all volunteer-level MPA results met the predefined acceptance criterion across both QC levels and storage temperatures (5/5 volunteers for each condition). The mean deviations from the nominal concentration across the individual volunteer samples ranged from −10.8 ± 1.2% to 1.9 ± 5.4% at 22 °C and from −12.3 ± 1.2% to 2.4 ± 3.4% at 6 °C. After 24 h of storage, the predefined acceptance criterion for MPA was met at 22 °C in 2/4 evaluable volunteers at the low-QC level and in 2/3 volunteers at the high-QC level. At 6 °C, none of the evaluable samples met the criterion at either QC level (0/3 for both low and high QC). In contrast, the acceptance criterion for MPAG after 12 h was met in 2/5 volunteers at the low-QC level at both 22 °C and 6 °C. At the high-QC level, the criterion was met in 1/5 volunteers at 22 °C and in 0/5 volunteers at 6 °C. After 24 h, the criterion for MPAG was met at 22 °C in 2/4 evaluable volunteers at the low-QC level and in 0/3 volunteers at the high-QC level. At 6 °C, none of the evaluable samples met the criterion at either QC level (0/3 for both low and high QC). For MPA, 12 h represented the longest tested pre-centrifugation storage interval at which the predefined acceptance criterion was met for every evaluated volunteer at both QC levels and storage temperatures. For MPAG, the criterion was not consistently met even at the earliest tested interval of 12 h; therefore, an acceptable pre-centrifugation storage interval for MPAG was not identified under the tested conditions. Volunteer-level percentage deviations are additionally summarized as medians and ranges in Supplementary Table S1. Blank cells in Table 3 indicate analyses that were not performed. Missing observations were not imputed.

## 4. Discussion

Saliva is an alternative matrix for TDM, particularly in pediatric patients, because it can be collected noninvasively and without the assistance of trained medical personnel [20]. This is especially relevant for drugs such as MPA, for which reliable pharmacokinetic assessment may require repeated sampling over several hours [1]. Home-based or minimally invasive saliva sampling has already been explored in several pediatric endocrine and nephrological settings [21,22,23]. In this context, the pre-analytical phase is particularly important. The usefulness of saliva-based monitoring depends not only on the analytical method, but also on the stability of the analyte in the collection device before centrifugation and laboratory processing. This problem is not specific to MPA. A critical review of saliva sampling methods indicated that relatively few studies adequately assess the impact of collection devices and sampling procedures on analyte recovery, concentration, and chemical form [24]. Therefore, the present study aimed to determine the maximum acceptable storage time for saliva samples collected using cotton Salivette^®^ devices, with MPA as the primary analyte of interest and MPAG as a secondary exploratory analyte.

Our previous studies addressed different analytical questions. The HPLC–FLD method was developed for MPA determination in saliva [17], whereas the LC–MS/MS method enabled simultaneous determination of MPA and MPAG in saliva samples from children with nephrotic syndrome [18]. In those studies, stability was evaluated mainly in processed saliva samples or under conditions related to analytical method validation. By contrast, the present study focuses on the pre-analytical period before centrifugation, when saliva remains in contact with the Salivette^®^ cotton swab. This distinction is important because analyte loss or apparent concentration changes may occur during contact with the sorbent material and may not be predicted from stability data obtained in processed filtrates.

The main finding of this study is that all volunteer-level MPA results met the predefined acceptance criterion after 12-h storage in non-centrifuged Salivette^®^ devices, at both room temperature and under refrigerated conditions. In contrast, MPAG did not consistently meet the predefined acceptance criterion in human saliva even after 12 h of storage. These findings suggest that saliva samples intended for MPA determination may be transported to the laboratory within the tested 12-h interval before centrifugation and further processing, provided that the storage temperature remains comparable to the evaluated conditions of 22 °C or 6 °C. For example, an evening sample collected at home could potentially be delivered and centrifuged in the laboratory the following morning, provided that the total pre-centrifugation interval does not exceed 12 h. However, this interpretation applies only when storage conditions remain comparable to the evaluated temperatures of 22 °C and 6 °C. Samples exposed to fluctuating or elevated temperatures, particularly above 25 °C during warmer seasons, should not be assumed to show comparable pre-analytical behavior. Moreover, because human saliva was evaluated only after 12 and 24 h, the exact maximum acceptable storage interval cannot be determined. The true threshold may lie between these time points and should be investigated using additional intermediate sampling times.

Inaccurate salivary concentrations caused by pre-analytical instability could lead to biased exposure estimates and weaken observed associations between MPA exposure, therapeutic response, and adverse effects [1,16]. This is particularly important in children with nephrotic syndrome, in whom inter-individual pharmacokinetic variability is substantial, and dose optimization may depend on reliable AUC-based assessment [10,25]. Standardized handling of saliva samples is therefore necessary to ensure the validity of concentration–time profiles before salivary measurements can be incorporated into limited-sampling models or prospective dose-individualization strategies [10,26]. Under standardized collection and storage conditions, serial salivary MPA concentrations could provide input data for the development of population pharmacokinetic or Bayesian models. In children with nephrotic syndrome, validated models could potentially support estimation of MPA AUC_0–12_ from a limited number of non-invasive samples. However, such an application would require prospective comparison with plasma exposure, definition of clinically relevant salivary targets, and external model validation.

An important observation was that PBS and artificial saliva produced more favorable results than authentic human saliva. In PBS, both MPA and MPAG met the predefined acceptance criterion for up to 24 h under both storage conditions. In artificial saliva, both analytes met the criterion for at least 24 h and, under some conditions, for up to 48 h. These findings indicate that results obtained only in buffer or artificial saliva may overestimate the acceptable pre-centrifugation interval and should not be directly extrapolated to clinical samples. Human saliva is a complex biological fluid containing enzymes, microorganisms, mucins, glycoproteins, electrolytes, metabolites, and food-derived residues. These components may influence analyte degradation, adsorption to the cotton swab, recovery after centrifugation, or ionization during LC–MS/MS analysis [24,27,28,29]. For this reason, the practical recommendation proposed in this study is based primarily on the results obtained in saliva from healthy volunteers, whereas PBS and artificial saliva served as supporting model matrices.

Previous studies have evaluated salivary MPA concentrations and their relationship with plasma exposure, but data on pre-analytical stability in saliva collection devices remain scarce [30]. Ferreira et al. [31] developed an LC–MS method for MPA and MPAG determination in oral fluid and plasma from kidney transplant recipients and suggested that oral fluid may be considered an alternative matrix for monitoring these analytes. Wiesen et al. [7] reported good recovery of MPA and MPAG from Salivette^®^ devices after 3 and 6 h of exposure. However, their study used synthetic swabs, whereas the present study evaluated cotton swabs. This difference is important because swab material may affect analyte adsorption and recovery. Similar effects have been described for other salivary analytes, for which collection devices, swab materials, and prolonged contact with the collection system may influence measured concentrations [24,32]. Compared with the shorter exposure times investigated previously, fulfillment of the acceptance criterion for MPA after 12-h provides a more practically relevant interval for the future evaluation of remote or home-based sampling.

The stability results for MPAG were less favorable and should be interpreted with caution. In several human saliva samples, MPAG concentrations appeared to increase rather than decrease during storage, indicating that the observed variability was not consistent solely with chemical degradation. The present study was not designed to determine the causes of these fluctuations, which may have included storage-dependent matrix changes, altered analyte recovery or ionization, interactions with the collection device, or other analytical and pre-analytical factors. The influence of absorbent saliva collection devices on measured concentrations is known to be analyte-dependent, highlighting the need to evaluate each analyte-device combination separately [24,33,34]. In our previous method-validation study, short-term apparent recovery from Salivette^®^ cotton swabs after a 5-min incubation at 37 °C ranged from 90.9% to 101.6% for MPA and from 100.1% to 100.7% for MPAG [18]. However, that experiment did not constitute a contemporaneous time-zero control for the present study and cannot exclude matrix- or time-dependent changes during prolonged storage of non-centrifuged devices. Published information on the pre-analytical stability of MPAG in saliva collected using cotton-based devices remains limited. Available data on the pre-analytical sensitivity of MPA glucuronide metabolites is derived mainly from plasma studies and therefore cannot be directly extrapolated to human saliva. Nevertheless, these studies provide indirect evidence that glucuronide metabolites may be sensitive to sample handling conditions [35,36]. Moreover, the occurrence of marked positive deviations also in PBS, where enzymatic and microbiological processes are unlikely, argues against attributing the apparent MPAG increases to a specific biological mechanism. We therefore do not interpret the positive deviations as evidence of true in-sample MPAG formation. Regardless of their underlying cause, MPAG results did not consistently meet the predefined acceptance criterion in human saliva under tested conditions. MPAG is the major pharmacologically inactive metabolite of MPA and is not currently an established primary target for saliva-based TDM. Although plasma MPAG may provide complementary pharmacokinetic information because of its relationship with MPA enterohepatic recirculation, renal function, and protein binding [1,37,38], these observations cannot be directly extrapolated to saliva. To date, no validated salivary MPAG therapeutic target or established relationship between salivary MPAG exposure and efficacy or toxicity has been demonstrated. MPAG was therefore evaluated as a secondary exploratory analyte rather than as an essential component of routine saliva-based TDM. Consequently, its inconsistent pre-analytical behavior does not undermine the potential utility of saliva for MPA monitoring. If MPAG determination is required for pharmacokinetic or research purposes, immediate centrifugation of the Salivette^®^ device and prompt sample processing would be advisable.

The ancillary frozen-storage experiment evaluated artificial-saliva filtrates that had been first stored in non-centrifuged Salivette^®^ devices for 12 or 24 h. After centrifugation, the resulting filtrates were subsequently stored at −80 °C for four months. Because matched measurements obtained immediately before freezing were not available, the effects of pre-centrifugation storage and subsequent frozen storage cannot be separated. The result therefore demonstrated only that the combined handling sequence did not consistently meet the predefined acceptance criterion, particularly for MPA; they do not establish that the observed deviations were caused specifically by storage at −80 °C. Moreover, because this experiment was limited to artificial saliva, the findings cannot be extrapolated to authentic human saliva or to filtrates obtained immediately after sample collection.

The operational ±15% acceptance criterion used in this study was selected with reference to the ICH M10 recommendations for QC samples above the LLOQ. The low QC concentration of 5 ng/mL was above the LLOQ of the LC–MS/MS method (2 ng/mL). However, because contemporaneous time-zero samples were not included, the present experiment did not constitute a formal stability validation fully consistent with the ICH M10 design. Fulfillment or non-fulfillment of the criterion therefore reflects deviations from the nominal concentrations rather than changes relative to a measured baseline. Nevertheless, results at 5 ng/mL require cautious interpretation, because the ±15% acceptance range corresponds to an absolute difference of only ±0.75 ng/mL. Thus, small absolute analytical or pre-analytical differences may result in relatively large percentage deviations, and isolated deviations at the low QC level should not be interpreted as evidence of a specific degradation mechanism. Syed and Srinivas [39] summarized substantial variability among published MPA, MPAG and mycophenolic acid acyl-glucuronide assays across biological matrices, including differences in sensitivity, precision, and accuracy. Therefore, the tested 12-h interval should be interpreted as the longest evaluated storage interval that met the predefined acceptance criterion for MPA under the investigated conditions, rather than as a broad safety margin or exact maximum acceptable storage time.

The study has several limitations. First, the experiments were performed using externally spiked saliva from only five healthy adult volunteers rather than authentic post-dose saliva samples from pediatric patients receiving MMF. Although spiking allowed the samples to be evaluated under controlled conditions and at predefined QC concentrations, saliva from pediatric patients may differ in composition because of age, disease state, concomitant medication, diet, oral hygiene, and variable salivary flow. Salivary flow rate, pH, protein content, mucin composition, oral microbiota and enzymatic activity may also differ between individuals and between adults and children. Therefore, the acceptability of the tested 12-h storage interval should be confirmed in saliva samples from children receiving MMF before implementation in pediatric TDM protocols. Nevertheless, the use of human saliva provided a more clinically relevant matrix than PBS or artificial saliva. Second, only cotton Salivette^®^ devices were evaluated. Cotton swabs were selected because they had been used in our previous salivary MPA studies, thereby ensuring continuity with the existing analytical protocol. Consequently, the present findings should not be extrapolated to synthetic Salivette^®^ swabs or other saliva collection systems. Direct comparison of cotton and synthetic collection materials during prolonged pre-centrifugation storage would be valuable in future studies. Third, storage was performed under controlled laboratory conditions at 22 °C and 6 °C, whereas home-based sampling may introduce additional variability, including inaccurate sampling time, recent food or beverage intake, delays in transport, and exposure to fluctuating temperatures. In particular, samples transported during warmer seasons may be exposed to temperatures above 25 °C. Because elevated and fluctuating temperatures were not evaluated, the present findings cannot be extrapolated to such conditions. In addition, only a standardized 5-min pre-storage incubation at 37 °C was evaluated. Longer in-mouth retention may occur in clinical or home-based sampling when an adequate saliva volume cannot be obtained within the recommended collection time. This could influence analyte recovery through prolonged contact with the cotton swab and stimulation-related changes in saliva volume and composition. Therefore, real-world feasibility and the effect of longer collection times should be evaluated in future studies. Fourth, no contemporaneous time-zero sample was prepared for each individual saliva matrix by applying the freshly spiked saliva to the cotton swab and centrifuging it immediately. The results were therefore evaluated relative to the nominal spiked concentrations rather than to measured baseline values. Consequently, the observed deviations may reflect not only storage-related changes, but also variability in sample preparation, swab recovery, matrix-dependent analytical response, or analytical measurement. Finally, evaporative loss was not assessed, and the total sample volume recovered after centrifugation was not systematically recorded. The contribution of water loss to the apparent concentration changes therefore cannot be excluded. In addition, the study was designed as an applied assessment of pre-centrifugation handling rather than as a mechanistic investigation. Degradation products, storage-dependent matrix effects, microbial activity, and time-dependent swab recovery were not specifically evaluated. Consequently, chemical degradation cannot be distinguished from other analytical or pre-analytical sources of variability, particularly in the case of MPAG.

Future studies should verify these findings in incurred post-dose saliva samples from pediatric patients receiving MMF and should directly compare cotton Salivette^®^ devices with synthetic swabs or other saliva collection systems under identical pre-centrifugation conditions. Studies including intermediate time points between 12 and 24 h would also be required to define the acceptable storage interval more precisely. In addition, the effects of fluctuating temperatures and temperature excursions representative of real-world transport, including exposure to temperatures above 25 °C, should be evaluated. Alternative microsampling strategies may also be worth considering. Recently, dried saliva collected using Volumetric Absorptive Microsampling has been proposed for MPA and MPAG pharmacokinetic assessment, suggesting that other sampling formats may improve pre-analytical robustness [40]. Finally, the most promising sampling approaches should be evaluated within population pharmacokinetic, Bayesian, or limited-sampling studies to determine whether they can reliably support salivary exposure estimation and future TDM in children.

## 5. Conclusions

In externally spiked saliva obtained from five healthy adult volunteers, MPA met the predefined acceptance criterion after 12 h of storage in non-centrifuged cotton Salivette^®^ devices at both room temperature (22 °C) and under refrigeration (6 °C). This finding supports the feasibility of a 12-h pre-centrifugation interval for future home-based or decentralized MPA sampling under comparable conditions. However, this interval represents the longest tested period at which all volunteer-level MPA results met the criterion and should not be interpreted as the exact maximum acceptable storage time. Results obtained in PBS and artificial saliva were more favorable than those obtained in human saliva, indicating that model matrices may overestimate acceptable pre-centrifugation storage. MPAG did not consistently meet the predefined acceptance criterion in human saliva even after 12 h; therefore, prompt centrifugation and processing are advisable when MPAG determination is required. Because the study used externally spiked saliva from only five healthy adults and did not include contemporaneous time-zero controls, the findings require confirmation using incurred post-dose samples from pediatric patients receiving MMF before implementation in clinical TDM or saliva-based dose-individualization strategies.

## Figures and Tables

**Table 1:** Stability assessment of MPA and MPAG in PBS after storage in Salivette^®^ devices at room temperature (22 °C) and under refrigerated conditions (6 °C) for up to 48 h. Results are expressed as percentage deviation from the nominal concentration.

Nominal Concentration	MPA		MPAG	
	24 h	48 h	24 h	48 h
	Room Temperature (22 °C)			
5 ng/mL	−5.7%	−24.2%	+5.4%	+93.9%
	−11.2%	−27.0%	−3.0%	+94.8%
	+2.7%	−26.8%	+3.5%	+73.4%
mean ± SD	−4.7 ± 7.0%	−26.0 ± 1.5%	+2.0 ± 4.4%	+87.4 ± 12.1%
500 ng/mL	−7.1%	−10.0%	−5.1%	+55.1%
	−5.5%	−14.9%	−5.8%	+78.5%
	−6.6%	−16.0%	−4.8%	+76.3%
mean ± SD	−6.4 ± 0.8%	−13.6 ± 3.2%	−5.2 ± 0.5%	+70.0 ± 12.9%
	**Refrigerator (6 °C)**			
5 ng/mL	−3.5%	−21.1%	+1.6%	+95.7%
	−12.3%	−29.2%	−6.9%	+39.9%
	−8.2%	−15.4%	−10.0%	+98.7%
mean ± SD	−8.0 ± 4.4%	−21.9 ± 6.9%	−5.1 ± 6.0%	+78.1 ± 33.1%
500 ng/mL	−9.0%	−10.7%	−0.9%	+65.5%
	+0.8%	−14.2%	−0.4%	+78.2%
	−7.3%	−14.6%	−1.3%	+77.5%
mean ± SD	−5.2 ± 5.2%	−13.2 ± 2.2%	−0.9 ± 0.4%	+73.7 ± 7.2%

Data are shown as individual replicate values and the mean ± SD from three technical replicates. The predefined acceptance criterion was met when the mean percentage deviation from the nominal concentration was within ±15%. Values outside this range indicate that the predefined acceptance criterion was not met. MPA, mycophenolic acid; MPAG, mycophenolic acid glucuronide; PBS, phosphate-buffered saline; SD, standard deviation.

**Table 2:** Stability assessment of MPA and MPAG in artificial saliva after storage in Salivette^®^ devices at room temperature (22 °C) and under refrigerated conditions (6 °C) up to 48 h. Results are expressed as percentage deviation from the nominal concentration.

Nominal Concentration	MPA				MPAG	
	12 h	24 h	48 h	12 h	24 h	48 h
	Room Temperature (22 °C)					
5 ng/mL	−2.4%	+3.2%	−7.7%	−0.4%	+5.1%	−6.7%
	−11.4%	+0.8%	−20.5%	+9.0%	+5.4%	−12.8%
	−10.7%	+0.2%	−18.3%	−13.0%	+3.4%	−7.3%
mean ± SD	−8.1 ± 5.0%	+1.4 ± 1.6%	−15.5 ± 6.8%	−1.5 ± 11.0%	+4.6 ± 1.1%	−9.0 ± 3.4%
500 ng/mL	−0.7%	−5.3%	−11.7%	+2.4%	−5.5%	−14.7%
	+1.9%	−7.9%	−13.5%	+2.7%	+0.5%	−14.7%
	−2.9%	−13.0%	−8.6%	+3.4%	−6.0%	−13.6%
mean ± SD	−0.6 ± 2.4%	−8.7 ± 3.9%	−11.3 ± 2.5%	+2.8 ± 0.5%	−3.7 ± 3.6%	−14.3 ± 0.7%
	**Refrigerator (6 °C)**					
5 ng/mL	−13.4%	−4.2%	−11.7%	−2.6%	+8.1%	−0.9%
	−13.4%	−10.2%	−9.9%	+14.6%	+5.0%	+12.6%
	−12.0%	−8.3%	−9.0%	−2.5%	+6.3%	−13.2%
mean ± SD	−12.9 ± 0.8%	−7.6 ± 3.1%	−10.2 ± 1.4%	3.2 ± 9.9%	+6.5 ± 1.5%	−0.5 ± 12.9%
500 ng/mL	−5.1%	−1.1%	−12.8%	+1.8%	+1.2%	−13.8%
	−4.4%	−4.5%	−6.3%	+3.3%	−2.3%	−14.1%
	−1.7%	−11.0%	−10.0%	+3.3%	−10.1%	−13.6%
mean ± SD	−3.7 ± 1.8%	−5.5 ± 5.1%	−9.7 ± 3.3%	+2.8 ± 0.9%	−3.8 ± 5.8%	−13.8 ± 0.2%

Data are shown as individual replicate values and the mean ± SD of three technical replicates. The predefined acceptance criterion was met when the mean percentage deviation from the nominal concentration was within ±15%. Values outside this range indicate that the predefined acceptance criterion was not met. MPA, mycophenolic acid; MPAG, mycophenolic acid glucuronide; SD, standard deviation.

**Table 3:** Stability assessment of MPA and MPAG in saliva from healthy volunteers after storage in Salivette^®^ devices at room temperature (22 °C) and under refrigerated conditions (6 °C) for up to 24 h. Results are expressed as percentage deviation from the nominal concentration.

Nominal Concentration	Volunteer No	MPA		MPAG	
		12 h	24 h	12 h	24 h
		Room Temperature (22 °C)			
5 ng/mL	1	−5.3 ± 4.7%	−9.1 ± 4.8%	**+15.9 ± 9.5%**	−4.9 ± 11.4%
	2	−10.8 ± 1.2%	−13.8 ± 1.2%	−1.4 ± 12.5%	**+30.4 ± 30.0%**
	3	−8.1 ± 3.4%	**−37.0 ± 17.6%**	**+16.9 ± 13.1%**	+12.4 ± 18.6%
	4	+0.3 ± 2.5%	**+22.6 ± 15.8%**	**+47.1 ± 26.9%**	**+41.0 ± 17.8%**
	5	−8.9 ± 2.2%		+6.6 ± 7.9%	
500 ng/mL	1	−7.0 ± 2.1%	−4.4 ± 3.0%	**+39.8 ± 5.7%**	**+28.9 ± 6.7%**
	2	−1.1 ± 1.9%	−13.8 ± 0.4%	**+28.5 ± 3.0%**	**+35.7 ± 10.1%**
	3	+1.9 ± 5.4%	**−15.5 ± 3.9%**	**+16.2 ± 10.0%**	**+39.2 ± 8.2%**
	4	−0.1 ± 1.6%		**+16.5 ± 13.0%**	
	5	−10.7 ± 2.9%		+5.7 ± 7.7%	
	**Refrigerator (6 °C)**				
5 ng/mL	1	−2.8 ± 2.2%	**−32.6 ± 6.5%**	**+35.4 ± 12.8%**	**+25.8 ± 27.5%**
	2	−5.3 ± 6.5%	**−24.6 ± 2.4%**	**+45.7 ± 21.4%**	**+69.3 ± 21.2%**
	3	−0.1 ± 2.1%	**+17.1 ± 16.2%**	**+41.1 ± 22.3%**	**+47.3 ± 33.1%**
	4	−12.3 ± 1.2%		+7.3 ± 5.5%	
	5	−4.0 ± 3.1%		+3.4 ± 5.7%	
500 ng/mL	1	−10.6 ± 1.4%	**−23.9 ± 6.5%**	**+51.0 ± 6.6%**	**+45.2 ± 8.1%**
	2	−8.7 ± 1.0%	**−27.7 ± 3.7%**	**+47.5 ± 17.7%**	**+29.7 ± 3.3%**
	3	+2.4 ± 3.4%	**−27.2 ± 3.8%**	**+61.9 ± 9.0%**	**+48.5 ± 2.7%**
	4	−1.1 ± 2.4%		**+26.0 ± 0.5%**	
	5	−1.8 ± 2.2%		**+25.5 ± 0.7%**	

Each volunteer-level value is presented as the mean ± SD of three technical replicates prepared from the same saliva sample. The five volunteers represent independent biological observations. Technical replicates were not treated as independent observations. Stability was assessed separately for each volunteer, and the predefined acceptance criterion was met when the mean percentage deviation from the nominal concentration was within ±15%. Values outside this range are shown in bold and indicate that the predefined acceptance criterion was not met. The number of evaluable volunteers may differ among conditions because some analyses were not performed; missing observations were not imputed. MPA, mycophenolic acid; MPAG, mycophenolic acid glucuronide.

## Data Availability

The data that support the findings of this study are available from the corresponding author upon reasonable request.

## Supplementary Materials

The following supporting information can be downloaded at: https://www.mdpi.com/article/10.3390/pharmaceutics18091106/s1, Table S1: Descriptive summary of percentage deviations from nominal MPA and MPAG concentrations in human saliva. Table S2: Long-term (four months) stability at −80 °C of MPA and MPAG in artificial saliva firstly stored for 12 and 24 h in Salivette^®^ devices. The stability is expressed as the percentage deviation from nominal concentration.

## Abbreviations

The following abbreviations are used in this manuscript:

AUC_0–12_	Area under the concentration-time curve from 0 to 12 h
HPLC–FLD	High-performance liquid chromatography method with fluorescence detection
LC–MS	Liquid Chromatography–Mass Spectrometry
LC–MS/MS	Liquid chromatography–tandem mass spectrometry
LLOQ	Lower limit of quantification
MMF	Mycophenolate mofetil
MPA	Mycophenolic acid
MPAG	Mycophenolic acid glucuronide
PBS	Phosphate-buffered saline
QC	Quality control
SD	Standard deviation
TDM	Therapeutic drug monitoring
