# Supplementary material for: Pre-Analytical Stability of Mycophenolic Acid and Its Glucuronide in Saliva Samples Stored in Cotton Salivette® Devices Before Centrifugation
Source: Pharmaceutics. 2026 Sep 2;18(9):1106. doi: 10.3390/pharmaceutics18091106 (PMC13610011; doi:10.3390/pharmaceutics18091106)
Supplement: Supplementary file 1 [file pharmaceutics-18-01106-s001.zip › pharmaceutics-4436382-supplementary.pdf]

Supplementary Table S1. Descriptive summary of percentage deviations from nominal MPA and MPAG concentrations in human saliva.

Analyte	Time	Temperature	QC level	Evaluable volunteers, N	Meeting criterion, n/N	Median deviation, %	Range, %
MPA	12 h	22°C	5 ng/mL	5	5/5	-8.1	-10.8 to 0.3
		22°C	500 ng/mL	5	5/5	-1.1	-10.7 to 1.9
		6°C	5 ng/mL	5	5/5	-4.0	-12.3 to -0.1
		6°C	500 ng/mL	5	5/5	-1.8	-10.6 to 2.4
	24 h	22°C	5 ng/mL	4	2/4	-11.5	-37.0 to 22.6
		22°C	500 ng/mL	3	2/3	-13.8	-15.5 to -4.4
		6°C	5 ng/mL	3	0/3	-24.6	-32.6 to 17.1
		6°C	500 ng/mL	3	0/3	-27.2	-27.7 to -23.9
MPAG	12 h	22°C	5 ng/mL	5	2/5	15.9	-1.4 to 47.1
		22°C	500 ng/mL	5	1/5	16.5	5.7 to 39.8
		6°C	5 ng/mL	5	2/5	35.4	3.4 to 45.7
		6°C	500 ng/mL	5	0/5	47.5	25.5 to 61.9
	24 h	22°C	5 ng/mL	4	2/4	21.4	-4.9 to 41.0
		22°C	500 ng/mL	3	0/3	35.7	28.9 to 39.2
		6°C	5 ng/mL	3	0/3	47.3	25.8 to 69.3
		6°C	500 ng/mL	3	0/3	45.2	29.7 to 48.5

MPA, mycophenolic acid; MPAG, mycophenolic acid glucuronide; QC, quality sample

Supplementary Table S2. Long-term (four months) stability at -80 °C of MPA and MPAG in artificial saliva firstly stored for 12 and 24 h in Salivette® devices. The stability is expressed as the percentage deviation from nominal concentration.

**Storage condition of filtrates**		**MPA**		**MPAG**	
		**5 ng/mL**	**500 ng/mL**	**5 ng/mL**	**500 ng/mL**
Room temperature		four months at -80 °C after 12 h in Salivette® devices			
		-32.9 %	-15.7 %	-26.0 %	+9.2 %
		-38.0 %	-13.8 %	+11.0 %	-12.1 %
		-39.0 %	-14.8 %	-21.4 %	-11.4 %
	mean ± SD	-36.6 ± 3.2 %	-14.8 ± 0.9 %	-12.1 ± 20.1 %	-4.8 ± 12.1 %
		four months at -80 °C after 24 h in Salivette® devices			
		-62.9 %	-22.1 %	-18.4 %	-16.1 %
		-51.7 %	-19.7 %	-5.9 %	-14.1 %
		-61.4 %	-21.8 %	+3.0 %	-15.2 %
	mean ± SD	-58.6 ± 6.1 %	-21.2 ± 1.3 %	-7.1 ± 10.7 %	-15.1 ± 1.0 %
Under refrigeration		four months at -80 °C after 12 h in Salivette® devices			
		-40.6 %	-14.4 %	+14.5 %	-3.6 %
		-32.2 %	-14.8 %	-19.7 %	-4.5 %
		-35.8 %	-11.9 %	-7.9 %	-4.0 %
	mean ± SD	-36.2 ± 4.2 %	-13.7 ± 1.6 %	-4.4 ± 17.3 %	-4.0 ± 0.5 %
		four months at -80 °C after 24 h in Salivette® devices			
		-75.2 %	-26.2 %	-17.6 %	-14.5 %
		-66.7 %	-24.6 %	-26.9 %	-14.8 %
		-77.7 %	-18.4 %	-20.2 %	-14.4 %
	mean ± SD	-73.2 ± 5.7 %	-23.1 ± 4.1 %	-21.6 ± 4.8 %	-14.6 ± 0.2 %

Data are shown as individual values and mean ± SD from three replicates. Stability was considered acceptable when the mean deviation from nominal concentration was within ±15%. Values outside this range indicate instability.

MPA, mycophenolic acid; MPAG, mycophenolic acid glucuronide
